# Supplementary material for: Prediction of plasma ctDNA fraction and prognostic implications of liquid biopsy in advanced prostate cancer
Source: Nat Commun. 2024 Feb 28;15:1828. doi: 10.1038/s41467-024-45475-w (PMC10902374; doi:10.1038/s41467-024-45475-w)
Supplement: Supplementary file 1 — Supplementary Information [file 41467_2024_45475_MOESM1_ESM.pdf]

Prediction of plasma ctDNA fraction and prognostic implications of liquid biopsy in advanced prostate cancer: **Supplementary Information**

Nicolette M. Fonseca[†1], Corinne Maurice-Dror[†2], Cameron Herberts[†1], Wilson Tu[1], William Fan[2], Andrew J. Murtha[1], Catarina Kollmannsberger[2], Edmond M. Kwan[1,2,3], Karan Parekh[1], Elena Schönlau[1], Cecily Q. Bernales[1], Gráinne Donnellan[1], Sarah W. S. Ng[1], Takayuki Sumiyoshi[1,4], Joanna Vergidis[5], Krista Noonan[6], Daygen L. Finch[7], Muhammad Zulfiqar[8], Stacy Miller[9], Sunil Parimi[2], Jean-Michel Lavoie[5], Edward Hardy[10], Maryam Soleimani[2], Lucia Nappi[1,2], Bernhard J. Eigl[2], Christian Kollmannsberger[2], Sinja Taavitsainen[11], Matti Nykter[11], Sofie H. Tolmeijer[1,12], Emmy Boerrigter[13], Niven Mehra[12], Nielka P. van Erp[13], Bram De Laere[14,15,16], Johan Lindberg[16], Henrik Grönberg[16], Daniel J. Khalaf[2], Matti Annala[1,11*], Kim N. Chi[1,2*], Alexander W. Wyatt[1,17*]

[1]Vancouver Prostate Centre, Department of Urologic Sciences, University of British Columbia, British Columbia, Canada; [2]Department of Medical Oncology, BC Cancer, Vancouver, British Columbia, Canada; [3]Department of Medicine, School of Clinical Sciences; Monash University; Melbourne, Victoria, Australia; [4]Department of Urology, Graduate School of Medicine, Kyoto University, Kyoto, Japan; [5]Department of Medical Oncology, BC Cancer, Victoria, British Columbia, Canada; [6]Department of Medical Oncology, BC Cancer, Surrey, British Columbia, Canada; [7]Department of Medical Oncology, BC Cancer, Kelowna, British Columbia, Canada; [8]Department of Medical Oncology, BC Cancer, Abbotsford, British Columbia, Canada; [9]Department of Radiation Oncology, BC Cancer, Prince George, British Columbia, Canada; [10]Tom McMurtry & Peter Baerg Cancer Centre, Vernon Jubilee Hospital, British Columbia Canada. [11]Prostate Cancer Research Center, Faculty of Medicine and Health Technology, Tampere University and Tays Cancer Center, Tampere, Finland; [12]Department of Medical Oncology, Research Institute for Medical Innovation, Radboud University, Nijmegen, The Netherlands; [13]Department of Pharmacy, Research Institute for Medical Innovation, Radboud University, , Nijmegen, The Netherlands; [14]Department of Human Structure and Repair, Ghent University, Ghent, Belgium; [15]Cancer Research Institute Ghent (CRIG), Ghent University, Ghent, Belgium; [16]Department of Medical Epidemiology and Biostatistics, Karolinska Institute, Stockholm, Sweden; [17]Michael Smith Genome Sciences Centre, BC Cancer, Vancouver, British Columbia, Canada

[†]These authors contributed equally to this work.
***These authors have jointly supervised this work.**

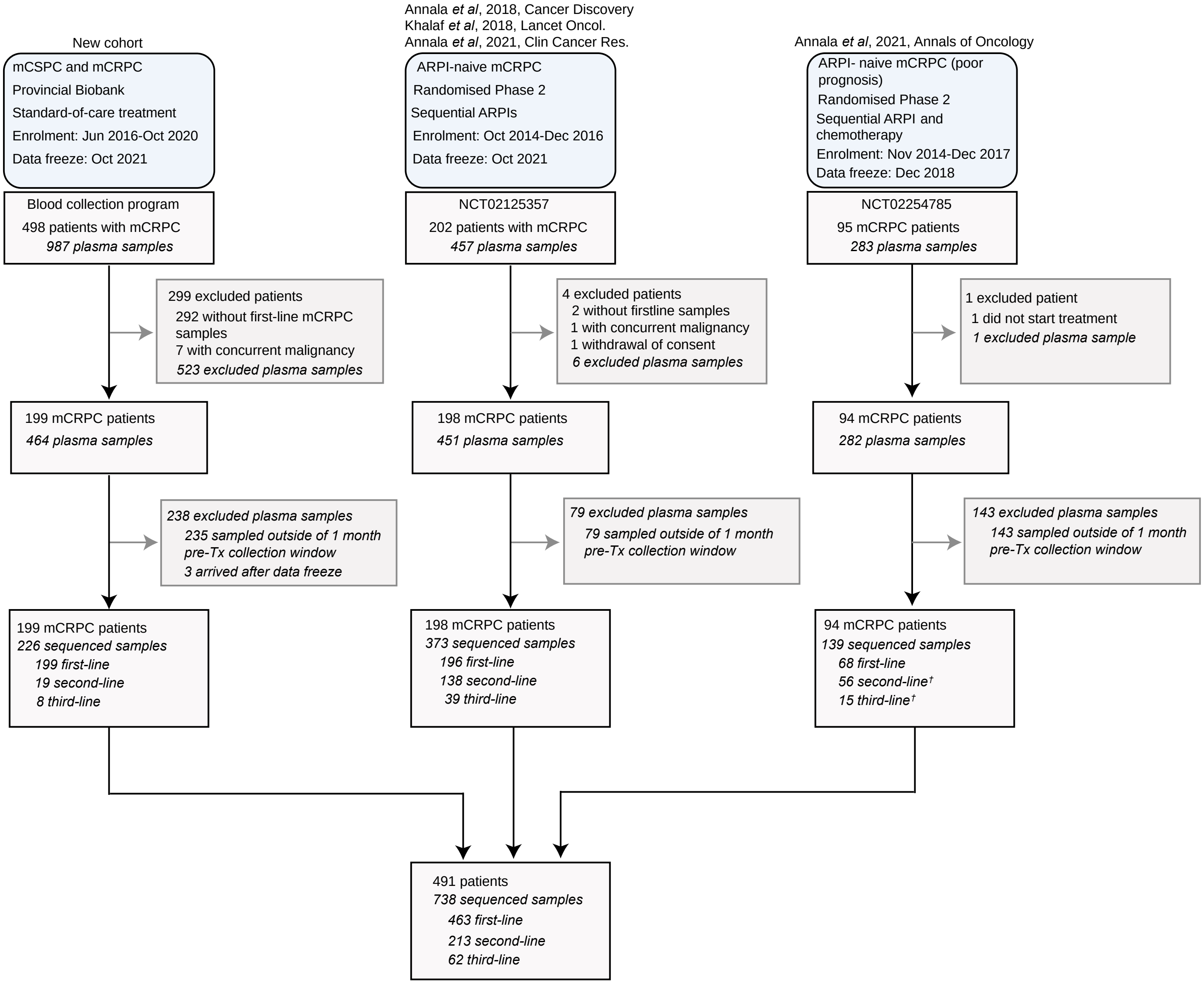

† Patients enrolled in OZM-054 were permitted to receive one prior course of docetaxel. 26 patients received one prior course of docetaxel prior to trial enrolment and therefore eligible on-trial samples collected from these patients were analysed as second-line (n = 26) or subsequently third-line at crossover (n = 15).

Supplementary Fig. 1. CONSORT flow diagram. Upright and italicized text refer to the number of available patients and samples at each step, respectively.

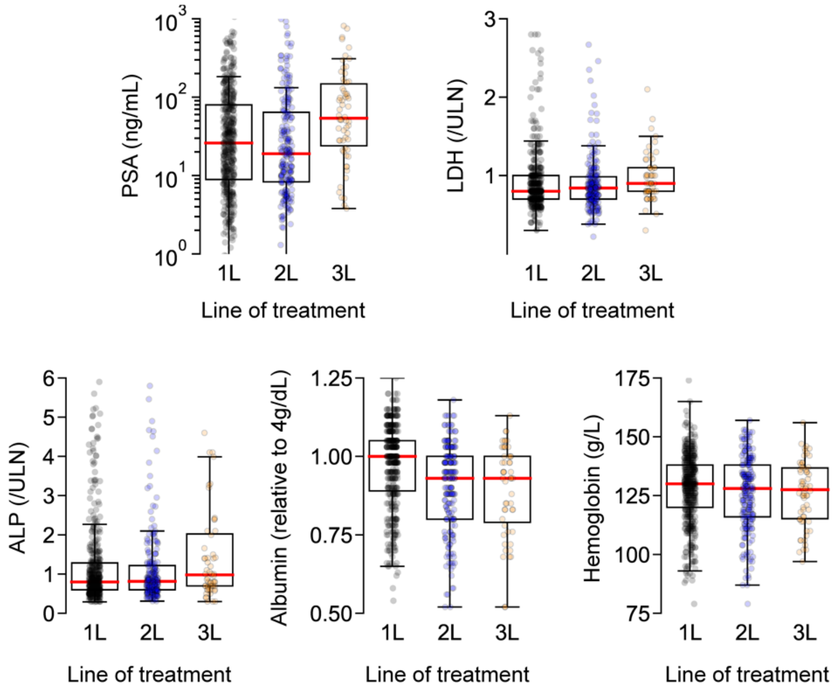

Supplementary Fig. 2. Per treatment-line distribution of additional clinical prognostic markers in our cohort. All variables are measured at time of line-specific mCRPC treatment initiation. See **Supplementary Data 3** for number of patients with evaluable data matched to each line of treatment.

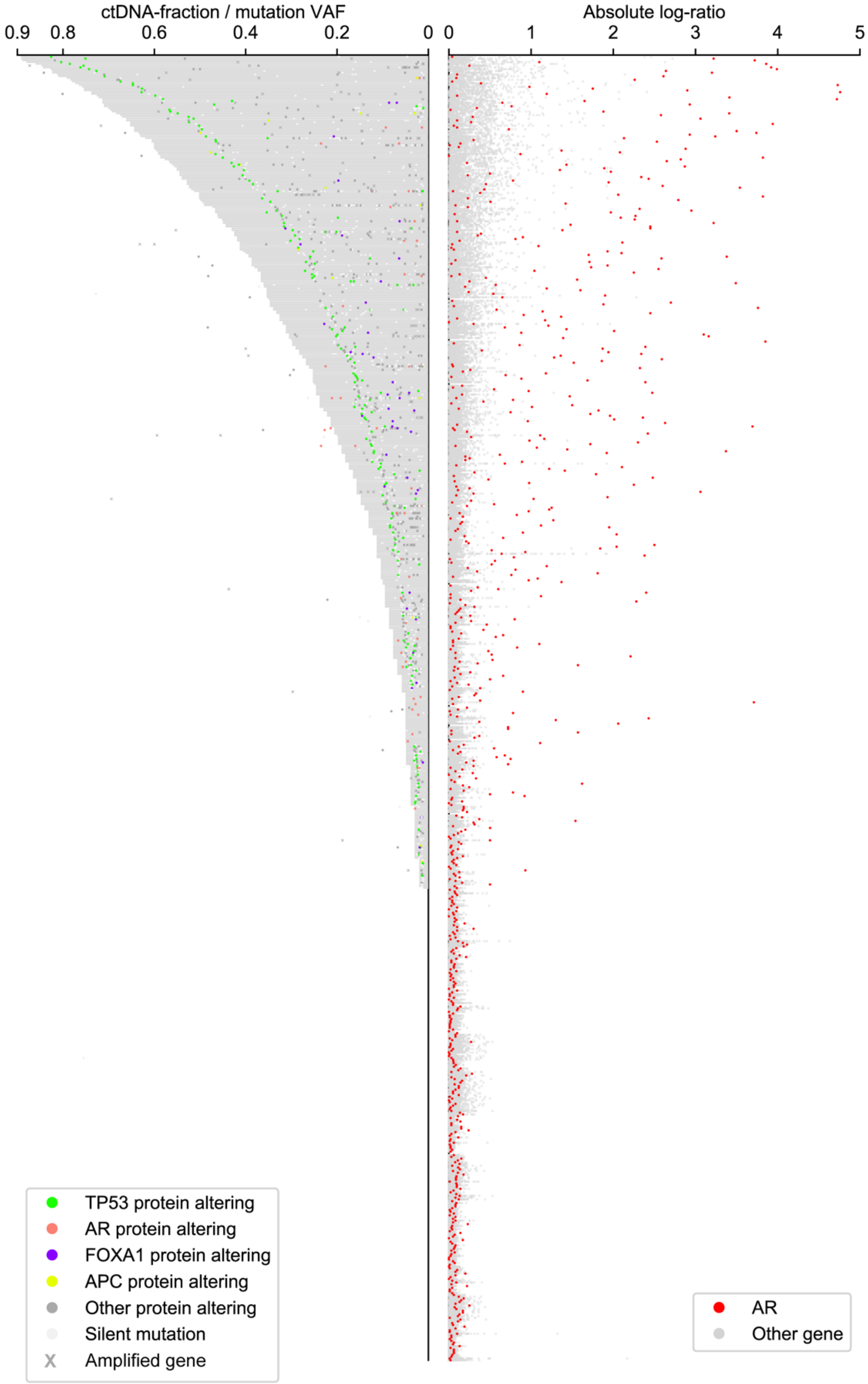

Supplementary Fig. 3. Mutation and copy-number evidence for ctDNA%. All somatic mutations, copy number alterations (via absolute log-ratio), and ctDNA fractions are plotted per sample (row). Mutations occurring on amplified genes are marked as X.

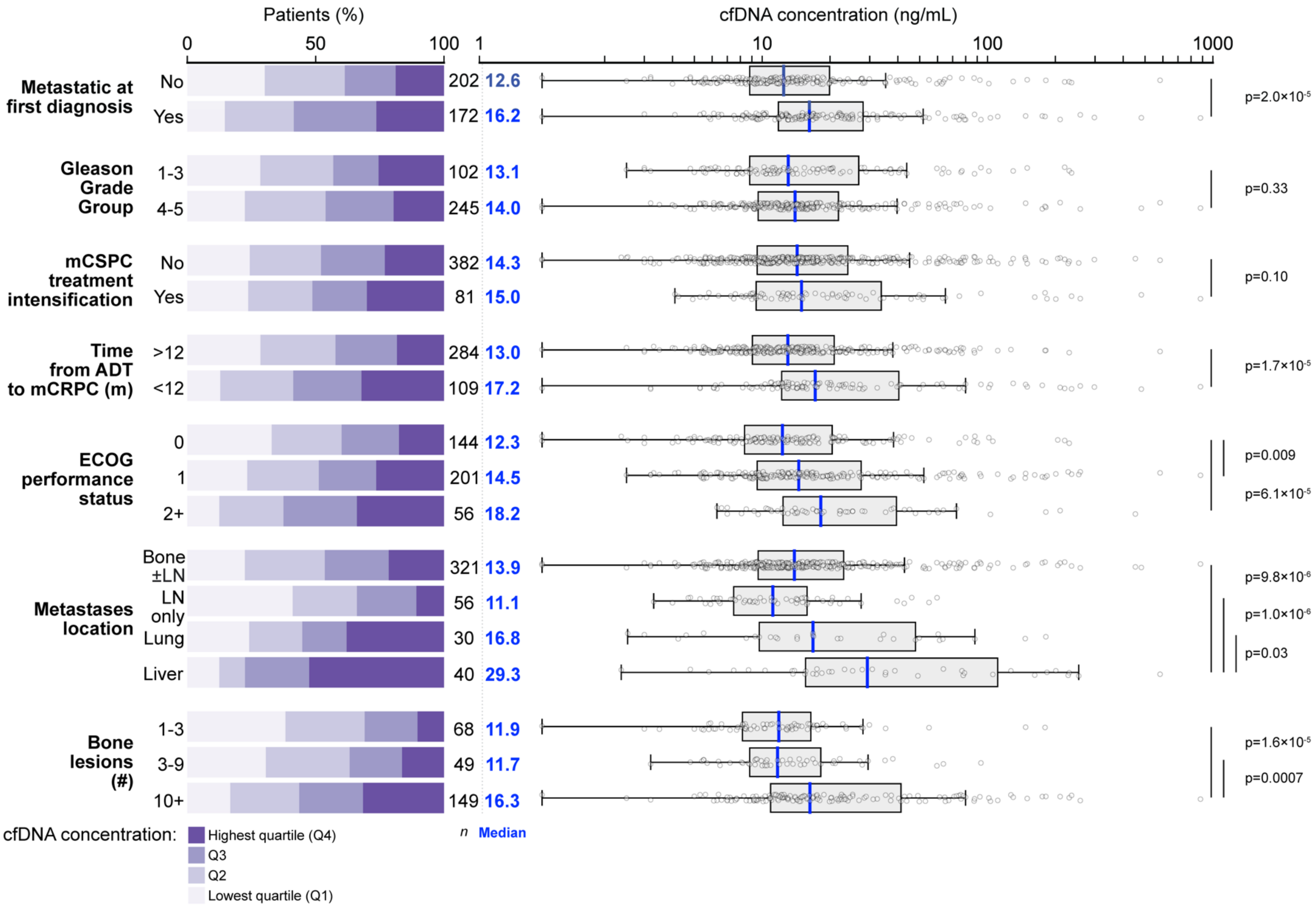

Supplementary Fig. 4. Serum and radiographic prognostic clinical features correlate with baseline cfDNA concentration. Fraction of patients per cfDNA concentration quartile (left) and cfDNA concentration as a continuous variable (right) across various categorical clinical subgroups (identical to those used in **Figure 2**).

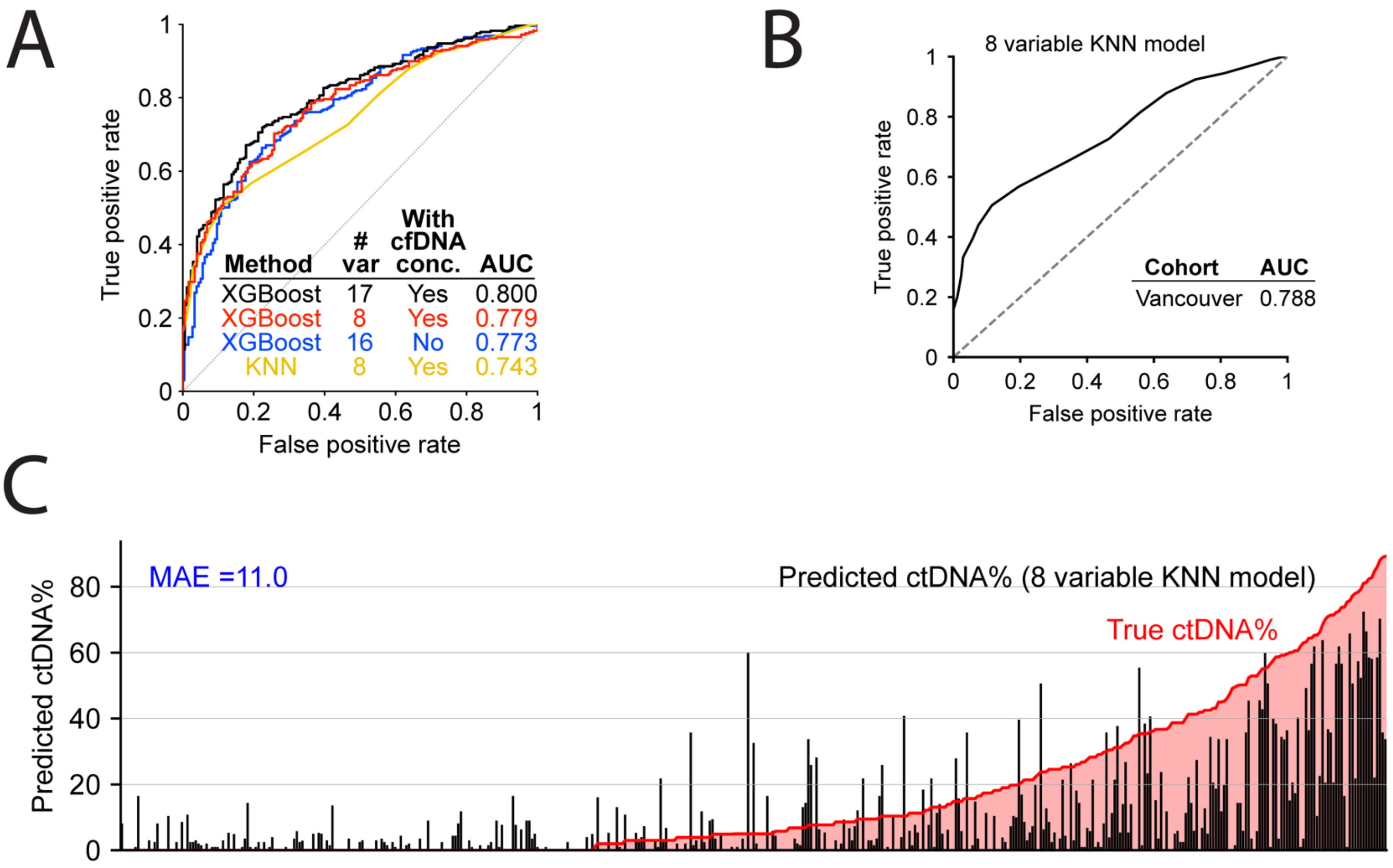

Supplementary Fig. 5. K-nearest neighbor prediction of ctDNA%. Receiver operating characteristic curves for (**a**) four separately trained and optimized XGBoost or K-nearest neighbor (KNN) prediction models evaluating different sets of clinical input features. (**b**) KNN prediction model with 8 routinely measured clinical variables. (**c**) KNN regression model with 8 clinical variables. Red curve shows ground-truth ctDNA% (i.e. from DNA sequencing). MAE = mean absolute error.

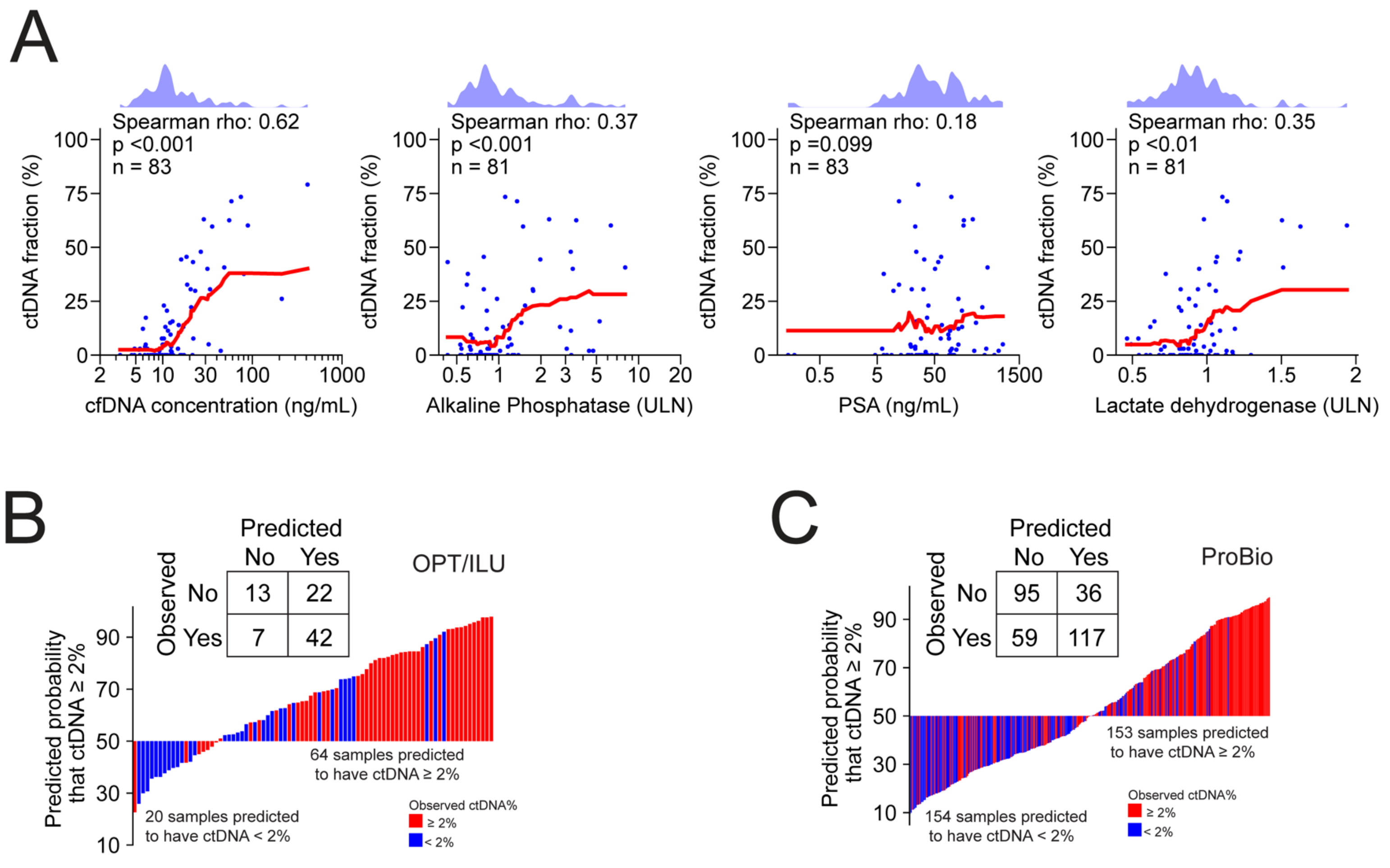

Supplementary Fig. 6. Validation of 8-feature XGBoost prediction of ctDNA%. (**a**) Correlation between ctDNA% and four continuous prognostic serum markers reported in the OPTIMUM & ILUMINATE trial datasets. K-nearest neighbor regression (neighbors=20 with uniform weights; red line) is used to nonparametrically visualize each bivariate relationship. Kernel density estimates shown above. (**b-c**) Predicted probability of ctDNA≥2% based on the 8-feature XGBoost model applied to the OPTIMUM & ILUMINATE (n=84) and ProBio (n=307) trial validation cohorts. True observed ctDNA≥2%status is indicated with color. In-set confusion matrix for classification of ctDNA≥2%.

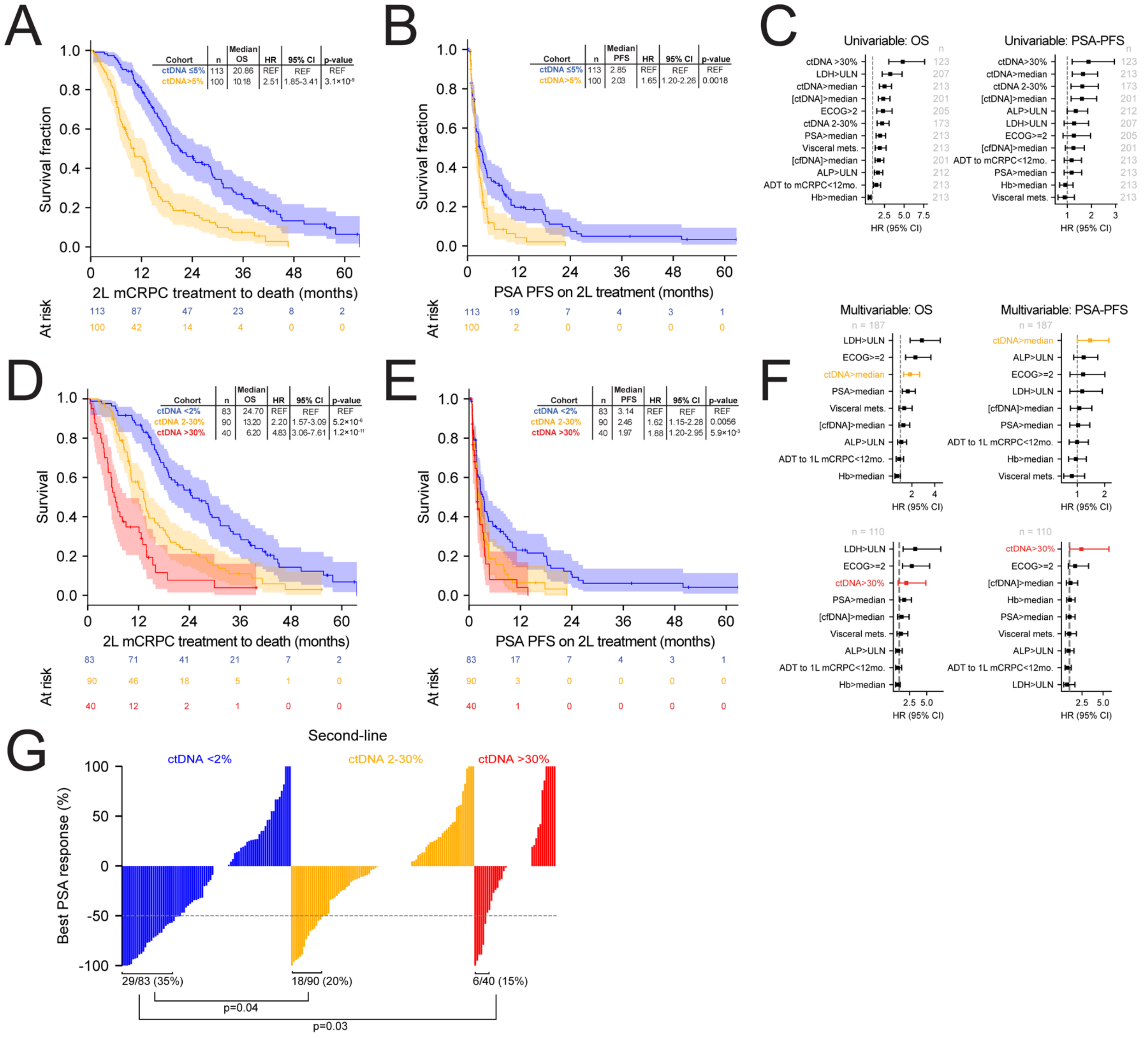

Supplementary Fig. 7. ctDNA% and clinical outcomes measured at initiation of second-line therapy. Kaplan-Meier estimates of time from initiation of second-line systemic therapy for mCRPC to death or last follow-up (**a**+**d**) and PSA progression-free survival on second-line therapy (**b**+**e**) stratified by synchronously-measured ctDNA% dichotomized by median (**a**+**b**) or by predefined bins (high, low, undetectable) (**d**+**e**). Shading indicates 95% confidence intervals; in-set tables show univariable HRs. Forest plots show HRs and 95% confidence intervals from univariable (**c**) and multivariable (**f**) Cox proportional hazard regression models incorporating ctDNA% plus additional clinical prognostic markers. (**g**) Waterfall plot showing best PSA response (relative to baseline PSA) on second-line mCRPC therapy stratified by baseline ctDNA% (ctDNA>30%, ctDNA 2-30%, and ctDNA<2%). P-values reflect Fisher's Exact Test's comparing the proportion of patients achieving a ≥50% PSA response across ctDNA categories.

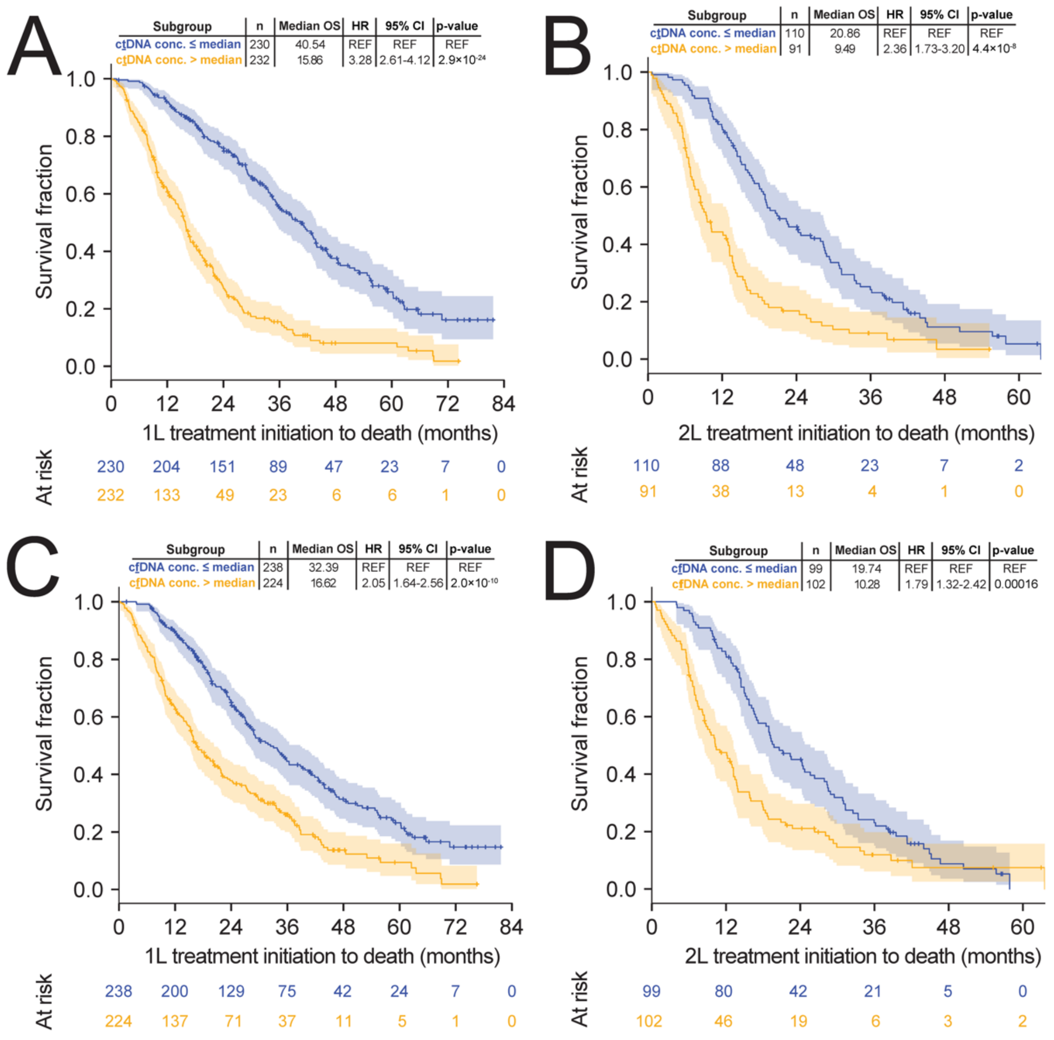

Supplementary Fig. 8. Prognostic relevance of baseline ctDNA and cfDNA concentration. Kaplan-Meier estimates of time from initiation of first and second-line systemic therapy for mCRPC to death or last follow-up stratified by synchronously measured ctDNA concentration (**a**+**b**) or cfDNA concentration (**c**+**d**) dichotomised by median. Shading indicates 95% confidence intervals.

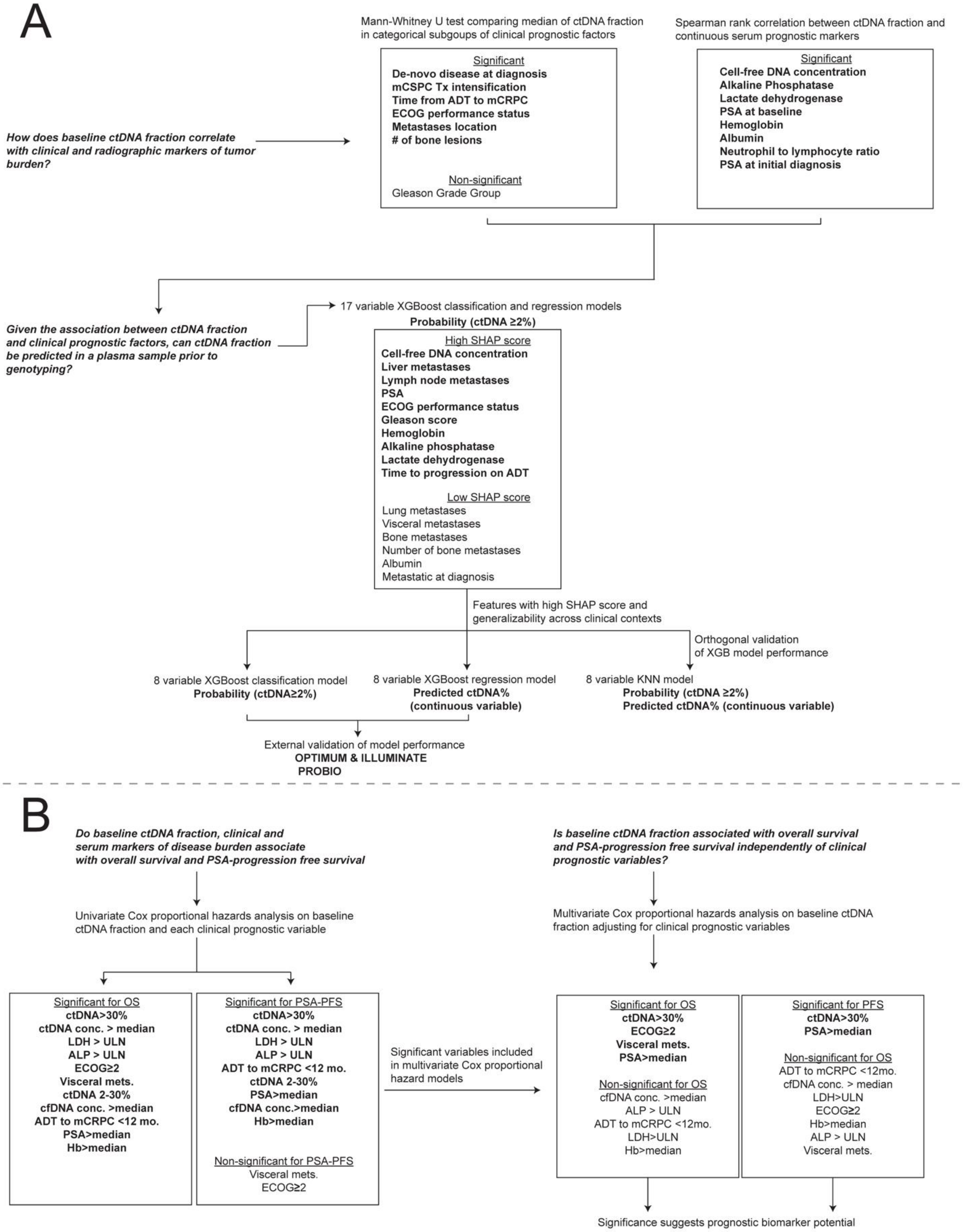

Supplementary Fig. 9. Statistical framework for evaluating the relationship between ctDNA fraction, clinical variables, and mCRPC survival outcomes. Flowchart showing the overarching experimental design and statistical approach for the manuscript's central objectives: (**a**) quantifying the association between ctDNA% and clinical markers of tumor burden and leveraging this information to develop a point-of-care ctDNA%-prediction tool, and (**b**) investigating the prognostic significance of measured ctDNA%.
