# Supplementary material for: Prediction of plasma ctDNA fraction and prognostic implications of liquid biopsy in advanced prostate cancer
Source: Nat Commun. 2024 Feb 28;15:1828. doi: 10.1038/s41467-024-45475-w (PMC10902374; doi:10.1038/s41467-024-45475-w)
Supplement: Supplementary file 3 — Description of Additional Supplementary Files [file 41467_2024_45475_MOESM3_ESM.pdf]

Description of Additional Supplementary Files

Supplementary Data Legends

Supplementary Data 1: List of clinical variables collected for all 491 mCRPC patients, grouped according to prostate cancer clinical setting.

Supplementary Data 2: Per patient/sample ctDNA fraction estimates.

Supplementary Data 3: Event rates for PSA-PFS and OS and summary of missing clinical data (per initiating line of therapy).

Supplementary Data 4: Univariable Cox proportional hazards model for overall survival and PSA-PFS. Hazard ratios, upper and lower 95% confidence intervals, and (unadjusted) p-values are displayed.

Supplementary Data 5: Multivariable Cox proportional hazard model for overall survival and PSA-PFS. Hazard ratios, upper and lower 95% confidence intervals, and (unadjusted) p-values are displayed.

Supplementary Data 6: Clinical variables used for training XGBoost and KNN models to predict ctDNA% and model performance metrics.
