# Supplementary material for: Prediction of plasma ctDNA fraction and prognostic implications of liquid biopsy in advanced prostate cancer
Source: Nat Commun. 2024 Feb 28;15:1828. doi: 10.1038/s41467-024-45475-w (PMC10902374; doi:10.1038/s41467-024-45475-w)
Supplement: Supplementary file 5 — Reporting Summary [file 41467_2024_45475_MOESM5_ESM.pdf]

nature portfolio

Corresponding author(s): Alexander W. Wyatt

Last updated by author(s): Dec 11, 2023

Reporting Summary

Nature Portfolio wishes to improve the reproducibility of the work that we publish. This form provides structure for consistency and transparency in reporting. For further information on Nature Portfolio policies, see our Editorial Policies and the Editorial Policy Checklist.

Statistics

For all statistical analyses, confirm that the following items are present in the figure legend, table legend, main text, or Methods section.

n/a	Confirmed	
☐	☒	The exact sample size (*n*) for each experimental group/condition, given as a discrete number and unit of measurement
☐	☒	A statement on whether measurements were taken from distinct samples or whether the same sample was measured repeatedly
☐	☒	The statistical test(s) used AND whether they are one- or two-sided<br>*Only common tests should be described solely by name; describe more complex techniques in the Methods section.*
☐	☒	A description of all covariates tested
☐	☒	A description of any assumptions or corrections, such as tests of normality and adjustment for multiple comparisons
☐	☒	A full description of the statistical parameters including central tendency (e.g. means) or other basic estimates (e.g. regression coefficient) AND variation (e.g. standard deviation) or associated estimates of uncertainty (e.g. confidence intervals)
☐	☒	For null hypothesis testing, the test statistic (e.g. *F*, *t*, *r*) with confidence intervals, effect sizes, degrees of freedom and *P* value noted<br>*Give P values as exact values whenever suitable.*
☒	☐	For Bayesian analysis, information on the choice of priors and Markov chain Monte Carlo settings
☒	☐	For hierarchical and complex designs, identification of the appropriate level for tests and full reporting of outcomes
☐	☒	Estimates of effect sizes (e.g. Cohen's *d*, Pearson's *r*), indicating how they were calculated

Our web collection on statistics for biologists contains articles on many of the points above.

Software and code

Policy information about availability of computer code

Data collection	No custom software was used to collect the data. Raw sequencing data was generated in-house. Sample meta-data was available in-house or provided by co-authors (e.g. manual review of patient electronic health records by clinical colleagues).
Data analysis	All statistical tests and data analyses were conducted in Python 3.7 (using pandas 0.25.0, numpy 1.16.4, scipy 1.6.2, and statsmodels 0.12.2) and Julia 1.5.1 (using HypothesisTests 0.10.9, and Distributions 0.25.53) languages. Visualizations were generated using matplotlib version 3.3.4 (Python). The following bioinformatics/genomic analysis software was used: cutadapt-1.11, seqkit-0.8, Bowtie-2.3.0, BWA (versions 0.7.15-r1140 and 0.7.17), samblaster-0.1.24, bedtools-2.25.0, samtools 1.12 (htslib 1.12), Picard 2.25.6, Mutato version 0.7 and ANNOVAR (version 20191024).<br><br>Custom computer code utilized for our machine-learning models is available on GitHub at https://github.com/annalam/ctdna-prediction-manuscript. Our complete ctDNA somatic variant calling pipeline is also available on GitHub at https://github.com/annalam/cfdna-wgs-manuscript-code. Comprehensive methodology including all new software (and versions) used is annotated within the manuscript.

For manuscripts utilizing custom algorithms or software that are central to the research but not yet described in published literature, software must be made available to editors and reviewers. We strongly encourage code deposition in a community repository (e.g. GitHub). See the Nature Portfolio guidelines for submitting code & software for further information.

Data

Policy information about availability of data

All manuscripts must include a data availability statement. This statement should provide the following information, where applicable:

- Accession codes, unique identifiers, or web links for publicly available datasets
- A description of any restrictions on data availability
- For clinical datasets or third party data, please ensure that the statement adheres to our policy

The human reference genome hg38 was downloaded from UCSC. Germline variant population frequency is available at gnomAD v3.0 (https://gnomad.broadinstitute.org/). De-identified sequencing data corresponding to patients enrolled in NCT02125357 have been deposited previously in the European Genome-Phenome Archive (EGA) database under the accession codes EGAS00001003113 and EGAS00001005318, and are available under standard EGA controlled release. EGA sequencing data corresponding to patients enrolled in the provincial blood biobanking program (i.e. new data generated in the context of this study) are available under the accession code [PENDING]. All other data supporting the findings of this study (i.e. source data) are available within the article (including its Supplementary Data).

Research involving human participants, their data, or biological material

Policy information about studies with human participants or human data. See also policy information about sex, gender (identity/presentation), and sexual orientation and race, ethnicity and racism.

Reporting on sex and gender	Sex and/or gender are not relevant for any findings in this study and were therefore not incorporated into study design, clinical data collection, nor execution of specific analyses. Prostate cancer only affects people born as biological males, and our cohort includes people with aggressive prostate cancer irrespective of gender identity. All samples are de-identified at time of collection, and all researchers are blind to patient gender identity and gender presentation.
Reporting on race, ethnicity, or other socially relevant groupings	Race, ethnicity, socioeconomic status, and other social variables were not collected and therefore not incorporated into the study analyses. We have acknowledged this as a limitation in our manuscript Discussion.
Population characteristics	All patients had metastatic castration-resistant prostate cancer (mCRPC). Patient characteristics are fully described in Table 1.
Recruitment	We analyzed patients who had initially enrolled on two prospective clinical trials (NCT02125357 and NCT02254785) and a province-wide prospective cfDNA biobanking program and had voluntarily consented to provide samples for research purposes.
Ethics oversight	University of British Columbia Ethics Board (certificate numbers provided within the manuscript document)

Note that full information on the approval of the study protocol must also be provided in the manuscript.

Field-specific reporting

Please select the one below that is the best fit for your research. If you are not sure, read the appropriate sections before making your selection.

☒ Life sciences ☐ Behavioural & social sciences ☐ Ecological, evolutionary & environmental sciences

For a reference copy of the document with all sections, see nature.com/documents/nr-reporting-summary-flat.pdf

Life sciences study design

All studies must disclose on these points even when the disclosure is negative.

Sample size	Sample size was not predetermined for this retrospective meta-analysis (no power calculations were performed). Blood samples from two prospective clinical trials (NCT02125357 and NCT02254785) and our province wide cfDNA biobank were included as long as the inclusion criteria were met (as specified in Methods) and the patient provided consent. We analyzed all possible samples within the constraints of: 1) the denominator of available patient/samples collected through these two clinical studies (NCT02125357 and NCT02254785) and the province-wide cfDNA biobank 2) our sample/patient eligibility criteria (detailed in our Methods). The exploratory and descriptive nature of our study and lack of pre-specified analyses means that target sample sizes were not possible; all statistical analyses are descriptive and effect sizes / p-values are reported as appropriate.
Data exclusions	The pre-specified inclusion criteria were those mandated as part of the original, previously-published, and publicly-available clinical studies (NCT02125357 and NCT02254785) from which our samples were sourced. Any cohort-level exclusions in the context of this current study were based on our patient inclusion criteria (which was not pre-specified; described in Methods): 1) All patients had histologically-confirmed prostatic adenocarcinoma (high-grade neuroendocrine and/or small-cell components were permitted), radiographic evidence of metastatic disease by conventional imaging (CT or bone scintigraphy), and castration-resistant prostate cancer defined as biochemical (PSA) or imaging progression despite castration levels of testosterone (PCWG3 criteria), 2) Plasma cfDNA samples must have been collected within 31 days prior to initiation of first, second, or third-line mCRPC treatment but not during active concurrent treatment (to minimize treatment-induced suppression of ctDNA quantities). 3) All patients must have provided at least a first-line mCRPC sample with exceptions made for 26 patients

in NCT02254785 who received one prior course of docetaxel and thus provided cfDNA samples associated with second line and/or third-line treatment. Patients with active concurrent malignancy were excluded.

Replication	All analyses are descriptive in nature. No experiments requiring technical or biological replicates were performed. Repeat sequencing of identical plasma or tissue DNA samples was not performed. Random seeds were used for all relevant computational/modelling analyses for reproducibility (documented in our online code repository). Select results were validated in external clinical trial cohorts.
Randomization	This retrospective meta-analysis did not directly incorporate randomisation, although the constituent clinical trials (NCT02125357 and NCT02254785) from which patients were accrued did involve random treatment assignment. Random permutation sampling was performed in the context of nested cross-validation for developing our ctDNA-fraction prediction tool. Clinical covariates were controlled for in survival outcome analysis using multivariate Cox proportional hazards modelling.
Blinding	Blinding was not performed because this is a retrospective exploratory meta-analysis without a pre-specified experimental plan. Investigators were not blinded to any patient data, patient allocation during experiments, or outcomes assessment.

Reporting for specific materials, systems and methods

We require information from authors about some types of materials, experimental systems and methods used in many studies. Here, indicate whether each material, system or method listed is relevant to your study. If you are not sure if a list item applies to your research, read the appropriate section before selecting a response.

Materials & experimental systems

n/a	Involved in the study	
☒	☐	Antibodies
☒	☐	Eukaryotic cell lines
☒	☐	Palaeontology and archaeology
☒	☐	Animals and other organisms
☐	☒	Clinical data
☒	☐	Dual use research of concern
☒	☐	Plants

Methods

n/a	Involved in the study	
☒	☐	ChIP-seq
☒	☐	Flow cytometry
☒	☐	MRI-based neuroimaging

Clinical data

Policy information about clinical studies

All manuscripts should comply with the ICMJE guidelines for publication of clinical research and a completed CONSORT checklist must be included with all submissions.

Clinical trial registration	NCT02125357 and NCT02254785
Study protocol	Patient inclusion criteria for this retrospective study are specified in the Methods.
Data collection	Patient samples were collected in the context of two clinical trials NCT02125357 and NCT02254785 and a province wide plasma cfDNA biobanking program. All samples were collected between October 2014 and October 2020.
Outcomes	Clinical endpoints evaluated in this study were overall survival (OS), prostate-specific antigen (PSA) progression free survival (PFS) and PSA response rate. PSA response was defined as ≥50% PSA decline from baseline. PSA-PFS (on first, second or third-line mCRPC therapy) was defined as the time from start of therapy to PSA progression or death. PSA progression was defined as an increase of at least 2 μg/L and ≥25% from nadir. For patients with no PSA decline, PSA progression was defined as an increase of ≥2 μg/L and ≥25% from baseline. Calculation of PSA progression did not require a subsequent confirmatory PSA measurement collected two weeks following initial PSA rise, although if a subsequent appropriate measurement was available, an additional PSA increase was required in order to meet progression. OS was defined as time from therapy initiation to time of death from any cause or last follow-up.

Plants

Seed stocks	Not relevant for our study.
Novel plant genotypes	Not relevant for our study.
Authentication	Not relevant for our study.
